# Maternal exercise represses Nox4 via SIRT1 to prevent vascular oxidative stress and endothelial dysfunction in SHR offspring

**DOI:** 10.3389/fendo.2023.1219194

**Published:** 2023-07-12

**Authors:** Yanyan Zhang, Meiling Shan, Xiaozhen Ding, Hualing Sun, Fang Qiu, Lijun Shi

**Affiliations:** ^1^ Department of Exercise Physiology, Beijing Sport University, Beijing, China; ^2^ Laboratory of Sports Stress and Adaptation of General Administration of Sport, Beijing Sport University, Beijing, China; ^3^ Key Laboratory of Physical Fitness and Exercise, Ministry of Education, Beijing Sport University, Beijing, China

**Keywords:** maternal exercise, endothelial function, histone deacetylase, hypertension, Nox4

## Abstract

Maternal exercise during pregnancy has emerged as a potentially promising approach to protect offspring from cardiovascular disease, including hypertension. Although endothelial dysfunction is involved in the pathophysiology of hypertension, limited studies have characterized how maternal exercise influences endothelial function of hypertensive offspring. In this study, pregnant spontaneously hypertensive rats and Wistar-Kyoto rats were assigned either to a sedentary lifestyle or to swimming training daily, and fetal histone deacetylase-mediated epigenetic modification and offspring vascular function of mesenteric arteries were analyzed. Maternal exercise ameliorated the impairment of acetylcholine-induced vasodilation without affecting sodium nitroprusside-induced vasodilation in mesenteric arteries from the hypertensive offspring. In accordance, maternal exercise reduced NADPH oxidase-4 (Nox4) protein to prevent the loss of nitric oxide generation and increased reactive oxygen species production in mesenteric arteries of hypertensive offspring. We further found that maternal exercise during pregnancy upregulated vascular SIRT1 (sirtuin 1) expression, leading to a low level of H3K9ac (histone H3 lysine 9 acetylation), resulting in the transcriptional downregulation of Nox4 in mesenteric arteries of hypertensive fetuses. These findings show that maternal exercise alleviates oxidative stress and the impairment of endothelium-dependent vasodilatation via SIRT1-regulated deacetylation of Nox4, which might contribute to improved vascular function in hypertensive offspring.

## Introduction

Hypertension is a major risk factor for the development of stroke, coronary heart disease, and renal disease (1). Endothelial dysfunction, in particular impaired endothelium-dependent relaxation, is commonly observed in patients and animal models of hypertension (2–4). Increased oxidative stress has been shown to play a central role in these pathological alterations in vascular endothelium in hypertension (5), however, the exact molecular mechanisms are not completely understood. An imbalance between prooxidative pathways and antioxidant system results in oxidative stress, which further leads to oxidant overproduction and exacerbates oxidative damage in hypertensive vascular (3). Increased reactive oxygen species (ROS), derived primarily from overactivated NADPH oxidases (Nox), are key signaling molecules regulating vascular dysfunction in hypertensive conditions (6). The Nox isoforms Nox1, Nox2, Nox4, and Nox5 have been described in the vasculature (7). Nox4 is highly expressed in endothelial cells (ECs) and it differs from other Nox family members by constitutively producing hydrogen peroxide (H_2_O_2_) instead of superoxide (8). Studies have proposed that Nox4 expression increases in the endothelium, mediating ROS production and vascular dysfunction in human arteries and mouse aortas during hypertension, in human skeletal muscle feed arteries during aging and type 1 diabetic mice (9–11). In contrast, endothelial Nox4 is described to enhance vasodilatation and reduce blood pressure (12–14). These inconsistent findings highlight a necessity for additional studies about Nox4 in ECs in hypertension.

Regular exercise has major benefits for individuals with hypertension, and there is strong epidemiological evidence that different types of exercise can prevent hypertension and are similarly effective in reducing blood pressure (SBP 8.7mmHg for endurance exercise, 7.2 mmHg for resistance exercise and 13.5 mmHg for combined exercise) (15, 16). Mounting evidence has documented that early-life exercise training is a positive stimulus for protecting offspring from hypertension, metabolic disorders or obesity (17–20). Our previous studies have shown that prenatal exercise improves vascular function and delays the development of hypertension in SHR offspring, which is associated with hypermethylation of Ca_V_1.2 channel α1_C_ gene in mesenteric arteries of fetuses and offspring (17, 21). However, whether maternal exercise can improve endothelial function in hypertensive offspring is unclear. More importantly, the role of endothelial Nox4 in vascular function and the fetal epigenetic reprogramming of Nox4 by exercise during pregnancy remain elusive. SIRT1 (sirtuin 1), one member of SIRTs (class III histone deacetylase) that are highly conserved NAD+-dependent deacetylases, plays a vital role in the pathogenesis of hypertension (22).

Here, we hypothesized that maternal exercise modulates oxidative stress via SIRT1/Nox4 signaling in mesenteric arteries to improve endothelial function in hypertensive offspring. By comparing spontaneously hypertensive rats (SHRs) with and without swimming exercise during pregnancy, we found that maternal exercise induces SIRT1 expression to inhibit Nox4 transcription by deacetylating histone H3K9 (histone H3 lysine 9) in mesenteric arteries during fetal development, thereby down-regulating Nox4-mediated signaling pathways to improve endothelium-dependent vasodilation in the adult offspring. Our studies may provide an insight into the epigenetic mechanism linking maternal exercise to fetal development and endothelial function, as well as long-term vascular health in hypertensive offspring. These findings may help us to identify prenatal exercise as a novel therapeutic approach to limit the inheritance of hypertension or other cardiovascular diseases to the next generation.

## Materials and methods

### Animals experiments

All procedures involving animals were reviewed and approved by the Animal Care and Use Committee of the Beijing Sport University. Wistar Kyoto rats (WKYs) and spontaneously hypertensive rats (SHRs) were obtained from Vital River Laboratory (Beijing, China) and housed in the temperature-controlled room under a 12-hour light-dark cycle. The 11-week-old virgin female WKYs and SHRs were mated overnight with 12-week-old males of the same strain. Determination of mating was confirmed by the presence of vaginal smears. The pregnant rats were randomly divided into four groups: WKY sedentary (p-WKY-SED), WKY exercised (p-WKY-EX), SHR sedentary (p-SHR-SED) and SHR exercised (p-SHR-EX) group. Maternal swimming exercise protocol was conducted as previously described with minor modifications (17). Briefly, maternal rats were subjected to swimming exercise training under a water depth of 40 cm at 34-35°C from gestation day 1 (GD 1) to GD 20. On the first day of training, rats from exercise groups swam for 20 min, with an additional 10 min/day applied on each subsequent day until 60 min. The pregnancy rats were exercised for 60 min until the end of GD20. The sedentary rats were placed in a cage containing water at a depth of 10 cm for the same duration as the exercising rats. At GD21, maternal rats were anaesthetized by 5% isoflurane inhalation and their uterus exposed. Fetuses were weighed, sexed and the mesenteric arteries were collected for further analyses. To examine the long-term effects of exercise during pregnancy, some maternal rats were prepared for delivering offspring. Body weight of these offspring (3-month-old, 3M) was measured and the blood pressure (BP) in conscious rats was acquired using the volume-pressure recording tail-cuff blood pressure monitoring system (CODA-6, Kent Scientific).

### Measurements of plasma oxidative stress biomarkers

Blood samples were collected in tubes containing EDTA as anticoagulant and centrifuged at 1500 rpm for 10 min at 4°C. Plasma malondialdehyde (MDA), superoxide dismutase (SOD) activity, glutathione peroxidase (GSH-PX) activity, and nitric oxide (NO) production were measured using the MDA assay kit, SOD assay kit, GSH-PX assay kit and NO assay kit, respectively. All assays were acquired from Nanjing Jiancheng Biological Engineering Co., Ltd and performed according to the manufacturer’s protocols.

### Arterial histology

Mesenteric arteries (second- to third-order) were dissected free of fat and connective tissue, and fixed with 4% paraformaldehyde for 12h. Paraffin-embedded tissue was sectioned at a 5-μm thickness. Sections were deparaffinized and then incubated with hematoxylin-and-eosin (H&E) staining. Images were acquired using an inverted microscope (IX71-F22PH, Olympus). Wall-to-lumen ratios were calculated as wall thickness/lumen diameter by using Image-Pro Plus.

### Measurement of vascular function

Isometric tension studies were performed on ~2 mm mesenteric artery (third-order) rings isolated from offspring rats in a wire myograph (Multi Myograph 620M, Danish Myo Technology). Briefly, the mesenteric artery segments were mounted on tungsten wires in a multi-channel myography containing of Krebs’ solution (mM, pH=7.4): 131.5 NaCl, 5 KCl, 1.2 NaH_2_PO_4_, 1.2 MgCl_2_, 2.5 CaCl_2_, 11.2 glucose, 13.5 NaHCO_3_ and 0.025 EDTA at 37°C, continuously gassed with 95% O_2_/5% CO_2_. After a 30 min equilibration period, the vessels viability and responses were tested using 60 mM KCl (*K*_max_). Endothelium-dependent vasodilatation evoked by acetylcholine (ACh, 10^-9^–10^-5^ M) and endothelium-independent vasorelaxation evoked by cumulative sodium nitroprusside (SNP, 10^-9^–10^-5^ M) were measured following pre-constricted with noradrenaline (NE, 10^-5^ M). In another experiment, to assess the involvement of NO and NADPH oxidase in endothelial dysfunction, ACh-induced vasodilatations were determined by the effect of L-NAME (10^-4^ M, NOS inhibitor) and Apocynin (3×10^-4^ M, NADPH oxidase inhibitor, 20 min before ACh) in NE-precontracted vessels, respectively. Data were acquired and analyzed using LabChart software (ADInstruments, Australia).

### Oxidative fluorescent microtopography

Endothelial superoxide production in frozen sections of rat mesenteric arteries was detected using the fluorescent probe dihydroethidium (DHE) (23). This superoxide indicator dye freely permeates the cell, and is oxidized to ethidium in the presence of O_2_^-^ (excitation/emission ~488/585 nm), which is intercalated within DNA. Briefly, mesenteric arteries were harvested and frozen in optimal cutting temperature compound (OCT). Cryosections (30-μm-thick) were cut at -25°C using a cryostat and placed on poly-lysine coated glass slide. Sections were incubated with DHE (10 μM, Invitrogen) for 5 min at 37°C in darkness. Sections were washed with ice-cold Krebs’ solution and then cover-slipped. Images were acquired using a laser-scanning confocal microscope and a ×63 oil objective (TCS-SP8, Leica, Germany). DHE fluorescence was quantified using ImageJ (NIH, USA) and measured only on the luminal side of the internal elastic lamina to quantify endothelial cell fluorescence. For each ring, mean fluorescence densities were calculated from the target region of the vessels to produce n=1.

### Real-time PCR

Total RNA was extracted from mesenteric arteries using the TRIzol Plus RNA Purification Kit (Invitrogen, USA) as instructed by the manufacturer, and first-strand cDNA was synthesized from 100-200 ng RNA. cDNA was amplified with Power SYBR Green PCR Master Mix using an ABI 7500 real-time PCR System. β-actin was used as an endogenous control. The primers used for the amplifications were as follows: Nox4, forward 5’-TTCTGGACCTTTGTGCCTATAC-3’, reverse 5’- ATCTGAGGGATGATTGATTACTG-3’; β-actin, forward 5’-CGCGAGTACAACCTTCTTGC-3’, reverse 5’-ATACCCACCATCACACCCTG-3’. Data were analyzed using the threshold cycle (Ct) relative quantification method (*ΔΔ*Ct). Expression for each gene was normalized to the level of β-actin transcript.

### Western blot

Western blot analysis was performed using as previously described (24). Briefly, mesenteric arteries were harvested and homogenized in ice-cold RIPA buffer with protease inhibitors. Equivalent amounts of protein (20 μg) were loaded onto 3−8% Tris-Acetate or 4−12% Bis-Tris gels (Invitrogen, USA) for electrophoresis and blotting. Membranes were incubated with the following primary antibodies: p-eNOS (1:2000, BD Biosciences), eNOS (1:2000, BD Biosciences), Nox4 (1:1000, Santa Cruz), SIRT1 (1:500, Cell Signaling Technology), H3K9ac (1:1000, Abcam), H3K14ac (1:1000, Abcam), H3 (1:1000, Abcam) and β-actin (1:2000, Santa Cruz) overnight at 4°C. Membranes were washed before incubating with horseradish peroxidase–conjugated secondary antibodies at room temperature. Immunoreactivity was visualized using enhanced chemiluminescence (ECL) and the Bio-Rad ChemiDOC XRS^+^ imaging system. Band intensity was quantified using ImageJ software.

### Chromatin immunoprecipitation-seq and qPCR

Mesenteric arteries from E21 embryonic fetuses were cross-linked with 1% formaldehyde for 15 min. The fixing reaction was stopped by adding 125 mM Glycine. After digesting and homogenizing, the chromatin was sonicated to generate DNA fragments 200-1000bp and 25 μL of sample lysate was used as input DNA. Primary antibodies against H3K9ac (ab10812, Abcam) or control normal rabbit IgG were captured on Dynabeads Protein G (Thermo Fisher Scientific) according to the manufacturer’s instructions. Chromatin fractions were immunoprecipitated with either Dynabeads-anti-H3K9ac or Dynabeads-IgG overnight at 4°C. The complexes were washed with buffers in the order of low salt buffer, high salt buffer, LiCl buffer, and TE buffer (10 mM Tris-HCl, 1 mM EDTA, pH 8.0), and precipitated in a magnetic separation rack. Cross-linking was digested with Proteinase K and ChIP Elution Buffer at 62°C for 2 hours. DNA was collected by phenol/chloroform/isoamyl alcohol extraction. Finally, DNA was dissolved in TE buffer and subjected to sequencing or qPCR.

For ChIP-seq, 10 ng each of ChIP or input DNA was sequenced on Illumina NovaSeq 6000 using NovaSeq 6000 S4 Reagent Kit according to the manufacturer’s instructions. The ChIP-seq assays raw data were quality checked using fastqc (http://www.bioinformatics.babraham.ac.uk/projects/fastqc/). Reads were aligned to rat genome (UCSC RN6) using BOWTIE software (V2.2.7). Next, peaks were called using the model-based analysis of ChIP-seq (v1.4.2) peak caller with default parameters and an FDR cut-off of 0.05. Peak-related genes were then identified. The immunoprecipitated DNA was subjected to PCR amplification employing primers surrounding a 146 bp region of Nox4 promoter (forward: 5′- CTTTGGAGTTTGACTCTTAACGT-3′; reverse: 5′- CCAGTGCACACCAGCTTAAT-3′). The enrichment ratio was calculated as: % INPUT = 2(Ct_input_ – Ct_ChIP_) × 100.

### Chemicals and statistical analysis

All chemical reagents were from Sigma-Aldrich unless otherwise stated. Values are expressed as mean ± SEM. GraphPad Prism software were used for statistical analyses. An unpaired, two-tailed Student *t*-test was used when two groups were compared, and two-way ANOVA with Sidak’s multiple comparisons test was performed to compare multiple groups. Statistical significance was defined as *P*<0.05.

## Results

### Effect of exercise during pregnancy on body weight and blood pressure in offspring

**Figure 1:**
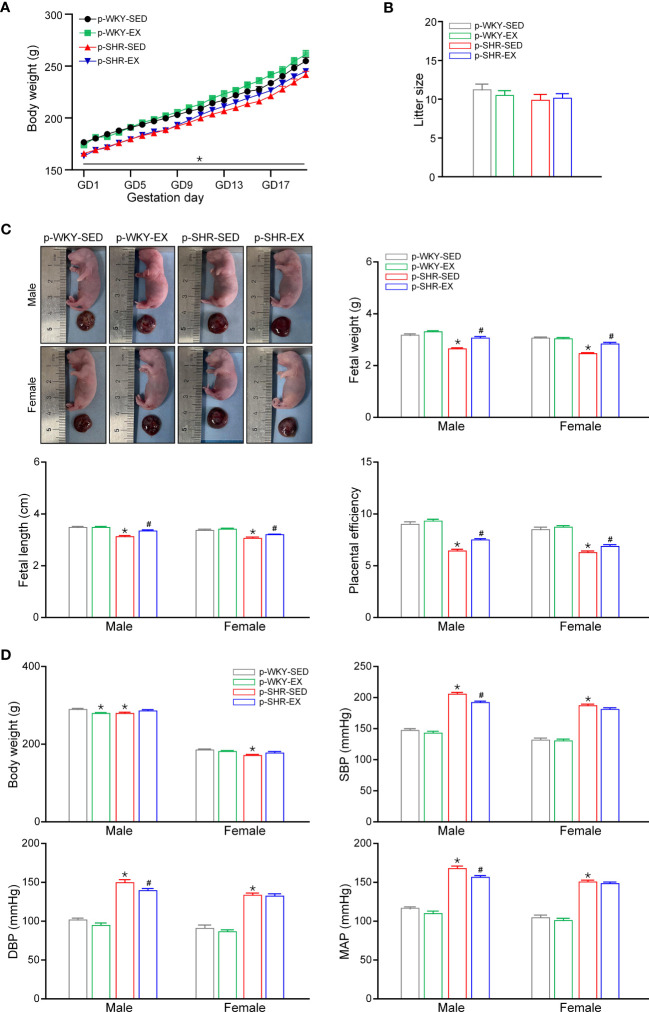
Effect of exercise during pregnancy on physiological parameters of offspring. **(A)** Daily body weight of pregnant rats (n=17-21). **(B)** Litter size of each group in ED21 fetuses (n=12-14). **(C)** Representative morphology of fetus and placenta in ED21 male and female offspring. Mean data of fetal weight, fetal length, and placental efficiency calculated as the ratio of fetal to placental weigh (n=30). **(D)** Body weight, SBP, DBP and MAP in 3M male and female offspring (n=15-30). * *P*<0.05 compared with p-WKY-SED; ^#^ *P*<0.05 compared with p-SHR-SED.

In the pregnant rats, the body weight in p-SHR-SED was significantly lower than in p-WKY-SED from GD1 to GD20 (all *P*<0.05). Maternal exercise during pregnancy did not change the body weight in exercised groups (**Figure 1A**). There was no significant difference of the liter size in any experimental group (**Figure 1B**).

As shown in **Figure 1C**, the fetal weight, fetal length and placental efficiency (the ratio of fetal to placental weights) in p-SHR-SED fetuses were significantly lower than in WKY-SED group (all *P*<0.05). In hypertensive groups, maternal exercise during pregnancy increased fetal weight, fetal length and placental efficiency in p-SHR-EX fetuses (all *P*<0.05). However, maternal exercise had no effect on fetal weight, fetal length and placental efficiency in WKY rats. In the 3M offspring, body weight was lower in both male and female in p-SHR-SED group than p-WKY-SED group. In WKY rats, maternal exercise decreased body weight in male offspring but not in female. Maternal exercise had no effect on body weight of 3M SHR offspring. SHR had a higher blood pressure than WKY offspring in both male and female by tail cuff plethysmography (**Figure 1D**). Maternal exercise reduced the systolic blood pressure (SBP: p-SHR-SED=205.36 ± 2.70 *vs.* p-SHR-EX=192.47 ± 1.78 mmHg, *P*<0.05), diastolic blood pressure (DBP: p-SHR-SED=150.36 ± 3.31 *vs.* p-SHR-EX=140.07 ± 2.00 mmHg, *P*<0.05) and mean arterial pressure (MAP: p-SHR-SED=168.37 ± 3.06 *vs.* p-SHR-EX=157.21 ± 1.76 mmHg, *P*<0.05) in 3M male offspring but had no effect on blood pressure in female offspring.

### Maternal exercise reduced plasma oxidative stress mediators and alleviated structural remodeling of mesenteric arteries from hypertensive offspring

**Figure 2:**
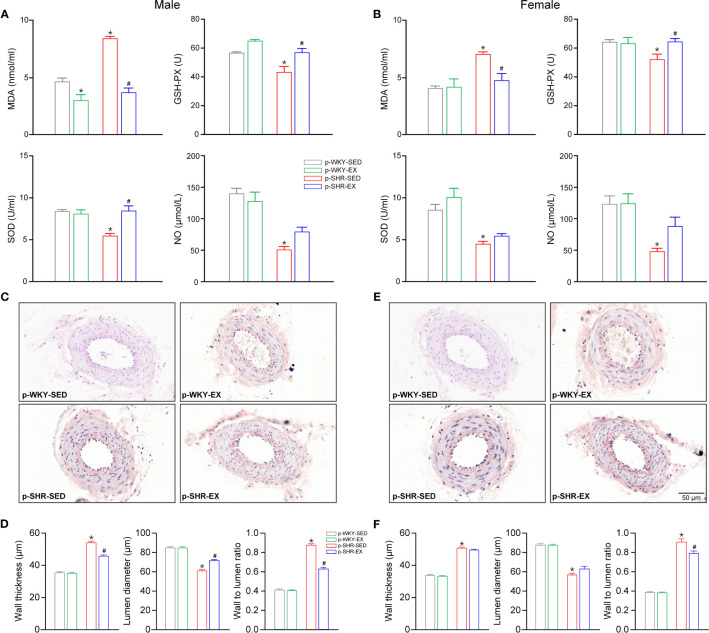
Effect of maternal exercise on biomarkers of oxidative stress and arterial morphology in offspring. **(A, B)** Plasma MDA content, GSH-PX activity, SOD activity, and NO production in 3M male **(A)** and female **(B)** offspring (n=6-18). **(C)** Representative images of H&E staining in mesenteric arteries from 3M male offspring. **(D)** Quantification analysis of wall thickness, lumen diameter and wall-to-lumen ratio of mesenteric arteries in 3M male offspring (n=6 in each group). **(E)** Representative images of H&E staining in mesenteric arteries from 3M female offspring. **(F)** Quantification analysis of wall thickness, lumen diameter and wall-to-lumen ratio of mesenteric arteries in 3M female offspring (n=6 in each group). * *P*<0.05 compared with p-WKY-SED; ^#^ *P*<0.05 compared with p-SHR-SED.

Plasma MDA levels were significantly increased in the p-SHR-SED group compared with the p-WKY-SED group, while maternal exercise decreased plasma MDA levels in the p-SHR-EX offspring (male: p-SHR-SED=8.41 ± 0.21 *vs.* p-SHR-EX=3.72 ± 0.42 nmol/ml, female: p-SHR-SED=7.07 ± 0.11 *vs.* p-SHR-EX=4.81 ± 052 nmol/ml; both *P*<0.05). Plasma GSH-Px and SOD activity were lower in the p-SHR-SED group than in the p-WKY-SED. Maternal exercise elevated plasma GSH-PX activity in both male and female offspring (male: p-SHR-SED=43.80 ± 4.20 *vs.* p-SHR-EX=57.41 ± 3.15 U, female: p-SHR-SED=52.81 ± 3.00 *vs.* p-SHR-EX=64.88 ± 1.71 U; both *P*<0.05), whereas increased SOD activity only in male offspring from p-SHR-EX group (male: p-SHR-SED=4.61 ± 0.22 *vs.* p-SHR-EX=5.59 ± 0.18 U/ml; *P*<0.05). Plasma NO levels decreased in the p-SHR-SED offspring and no changes in plasma NO levels were observed in p-SHR-EX group (**Figures 2A, B**).

The second-order mesenteric arteries from both male and female p-SHR-SED offspring exhibited increased wall thickness and artery wall-to-lumen ratio, and decreased lumen diameter (all *P*<0.05). As shown in **Figures 2C, D**, maternal exercise alleviated structural remodeling by reducing wall thickness (p-SHR-SED: 54.02 ± 0.61 *vs.* p-SHR-EX: 45.53 ± 0.70 μm) and artery wall-to-lumen ratio (p-SHR-SED: 0.88 ± 0.01 *vs.* p-SHR-EX: 0.63 ± 0.02), and increasing lumen diameter (p-SHR-SED: 61.18 ± 1.27 *vs.* p-SHR-EX: 71.87 ± 1.06 μm) in mesenteric arteries from p-SHR-EX group (all *P*<0.05). In contrast, in female offspring, maternal exercise enhanced artery wall-to-lumen ratio but did not change the wall thickness and lumen diameter (p-SHR-SED: 0.91 ± 0.03 *vs.* p-SHR-EX: 0.79 ± 0.03, *P*<0.05; **Figures 2E, F**).

### Maternal exercise ameliorated endothelium-dependent relaxation of mesenteric arteries from hypertensive offspring

**Figure 3:**
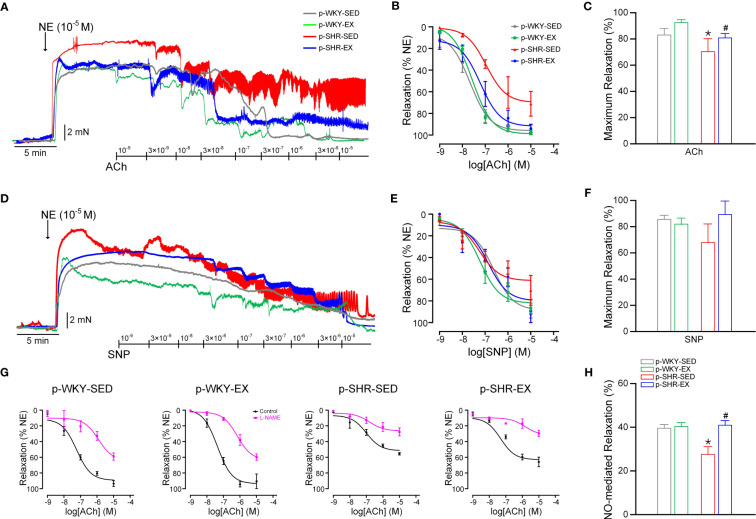
Effect of maternal exercise on vasodilatation function in mesenteric arteries from 3M male offspring. **(A)** Original traces of concentration-dependent vasodilatation evoked by acetylcholine (ACh, 10^-9^–10^-5^ M). Mesenteric artery rings were contracted with NE (10^-5^ M). **(B)** Corresponding concentration–response curves for endothelial-dependent vasodilatation to ACh. **(C)** Maximum relaxation (%) in response to 10^-5^ M ACh. **(D)** Original traces of concentration-independent vasodilatation evoked by sodium nitroprusside (SNP, 10^-9^–10^-5^ M). **(E)** Corresponding concentration–response curves for endothelial-independent vasodilatation to SNP. **(F)** Maximum relaxation (%) in response to 10^-5^ M SNP. **(G)** Endothelium-dependent vasodilatations to ACh were determined in the absence and presence of L-NAME (10^-4^ M, NOS inhibitor). **(H)** Contribution of NO-derived vasodilators in mesenteric arteries of 3M male offspring (n=3-6 animals per group). * *P*<0.05 compared with p-WKY-SED; ^#^ *P*<0.05 compared with p-SHR-SED.

**Figure 4:**
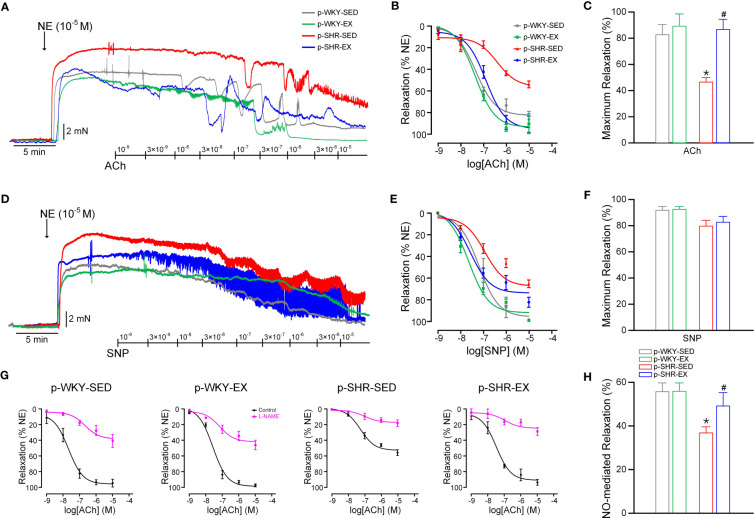
Effect of maternal exercise on vasodilatation function in mesenteric arteries from 3M female offspring. **(A)** Original traces of concentration-dependent vasodilatation evoked by acetylcholine (ACh, 10^-9^–10^-5^ M). Mesenteric artery rings were contracted with NE (10^-5^ M). **(B)** Corresponding concentration–response curves for endothelial-dependent vasodilatation to ACh. **(C)** Maximum relaxation (%) in response to 10^-5^ M ACh. **(D)** Original traces of concentration-independent vasodilatation evoked by sodium nitroprusside (SNP, 10^-9^–10^-5^ M). **(E)** Corresponding concentration–response curves for endothelial-independent vasodilatation to SNP. **(F)** Maximum relaxation (%) in response to 10^-5^ M SNP. **(G)** Endothelium-dependent vasodilatations to ACh were determined in the absence and presence of L-NAME (10^-4^ M, NOS inhibitor). **(H)** Contribution of NO-derived vasodilators in mesenteric arteries of 3M female offspring (n=3-4 animals per group). * *P*<0.05 compared with p-WKY-SED; ^#^ *P*<0.05 compared with p-SHR-SED.

Next, we investigated the effects of maternal exercise on endothelium dependent and independent relaxation of mesenteric arteries by isometric tension studies in offspring. The endothelium-dependent relaxation induced by ACh in NE-contracted mesenteric arteries was reduced in p-SHR-SED compared with p-WKY-SED and maternal exercise improved this relaxation in both male and female offspring (maximum relaxation: p-SHR-SED=70.58 ± 9.84 *vs.* p-SHR-EX: 81.09 ± 3.14%, **Figures 3A–C**; p-SHR-SED=46.60 ± 3.13 *vs.* p-SHR-EX: 86.65 ± 7.44%, **Figures 4A–C**; both *P*<0.05). Endothelium-independent vasodilatation in response to SNP was similar among groups in both male (**Figures 3D–F**) and female (**Figures 4D–F**) offspring.

We next determined the effects of maternal exercise on the relative contribution of the NOS-derived NO-mediated vasodilatation in mesenteric arteries by using the NOS inhibitor L-NAME. L-NAME incubation inhibited ACh relaxation in mesenteric arteries from all experimental groups (**Figures 3G**, **4G**), indicating that NO-derived vasodilator plays an important role in modulating vascular function in mesenteric arteries of both male and female offspring. The relative contribution of NOS-derived vasodilator (observed in the presence of L-NAME) was markedly reduced in mesenteric arteries of p-SHR-SED offspring compared to p-WKY-SED control offspring (**Figures 3H**, **4H**), further suggesting the loss of eNOS-derived NO is responsible for the impairment in p-SHR-SED mesenteric arteries. In contrast, maternal exercise markedly ameliorated NO-derived vasodilation in p-SHR-EX group, which leads to endothelial-protective effects in hypertensive offspring (male: p-SHR-SED=27.77 ± 3.26 *vs.* p-SHR-EX: 41.00 ± 1.91%, female: p-SHR-SED=37.23 ± 2.85 *vs.* p-SHR-EX: 49.56 ± 5.95%; both *P*<0.05).

### Maternal exercise increased NO generation and decreased ROS production in mesenteric arteries from hypertensive offspring

**Figure 5:**
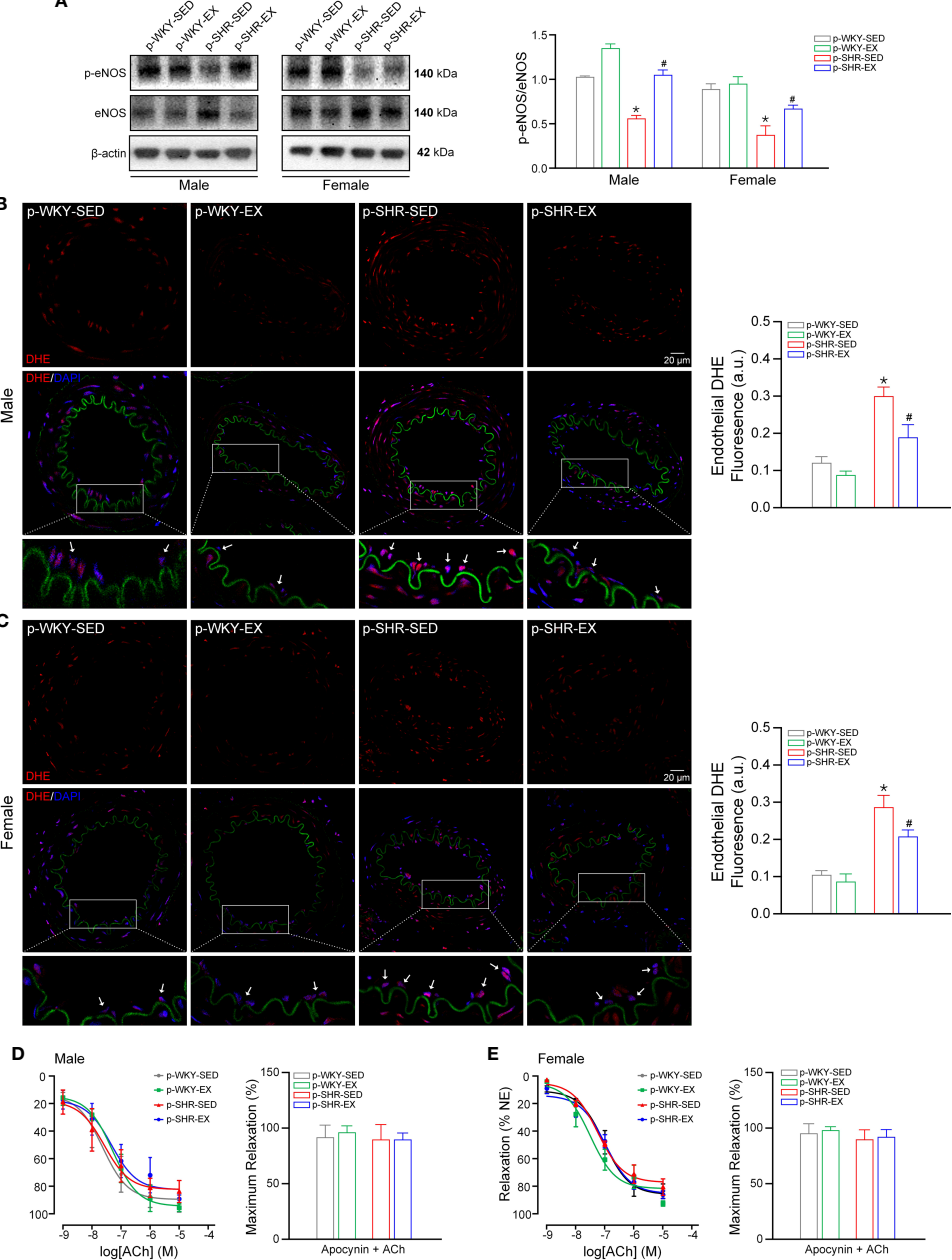
Effect of maternal exercise on NO bioactivity and ROS generation in mesenteric arteries from 3M male and female offspring. **(A)** Representative immunoblots showing phosphorylation of eNOS at Ser1177, eNOS and β-tubulin in mesenteric arteries from male and female offspring. Quantification data measured as band density of p-eNOS/total eNOS protein (n=6 animals per group). **(B, C)** DHE fluorescence to measure in endothelial ROS production in frozen sections of second-order mesenteric arteries from 3M male **(B)** and female **(C)** offspring. Representative mesenteric sections (×63) showing red endothelial cells with blue DAPI (arrows). Endothelium-derived ROS production, quantified in arbitrary units as area of luminal red staining/length of luminal surface. **(D, E)** Concentration–response curves for endothelial-dependent vasodilatation to ACh and maximum relaxation (%) in response to 10^-5^ M ACh in the presence of Apocynin (3×10^-4^ M, NADPH oxidase inhibitor) in mesenteric arteries from male **(D)** and female **(E)** offspring (n=3-5 animals per group). * *P*<0.05 compared with p-WKY-SED; ^#^ *P*<0.05 compared with p-SHR-SED.

To examine whether NO production contributes to improved NO-derived vasodilation by maternal exercise, phosphorylation of eNOS (p-eNOS) and total eNOS protein levels were assessed by Western blot in mesenteric arteries from 3M offspring. In p-SHR-SED offspring, the p-eNOS relative to the total eNOS protein content was significantly decreased in mesenteric arteries compared with p-WKY-SED controls (male: p-WKY-SED=1.03 ± 0.01 *vs.* p-SHR-SED: 0.57 ± 0.03, female: p-WKY-SED=0.90 ± 0.05 *vs.* p-SHR-SED: 0.38 ± 0.10; both *P*<0.05). Maternal exercise increased the p-eNOS/eNOS level nearly 1.9-fold and 1.8-fold in mesenteric arteries of hypertensive male and female offspring, respectively, but not in normotensive offspring (**Figure 5A**).

We next assessed endothelial generation of ROS (including superoxide and hydrogen peroxide), by DHE fluorescence in frozen sections of second-order mesenteric arteries. In both male and female offspring, we observed that p-SHR-SED mesenteric arteries generated nearly 3-fold more endothelium-derived ROS production than p-WKY-SED controls. After maternal exercise, the levels of endothelium-derived ROS production were significantly reduced in mesenteric arteries from p-SHR-EX offspring when compared with p-SHR-SED. However, the endothelium-derived ROS production was unchanged in p-WKY-EX offspring mesenteric arteries compared with p-WKY-SED (**Figures 5B, C**). Taken together, these data demonstrate that maternal exercise increases NO production and reduces ROS production in mesenteric arteries, leading to an enhancement of eNOS-derived NO-mediated vascular tone and improved endothelium-dependent vasodilatation in hypertensive offspring.

### Maternal exercise induces H3K9 deacetylation of Nox4 promoter via activating SIRT1 in hypertensive fetuses

**Figure 6:**
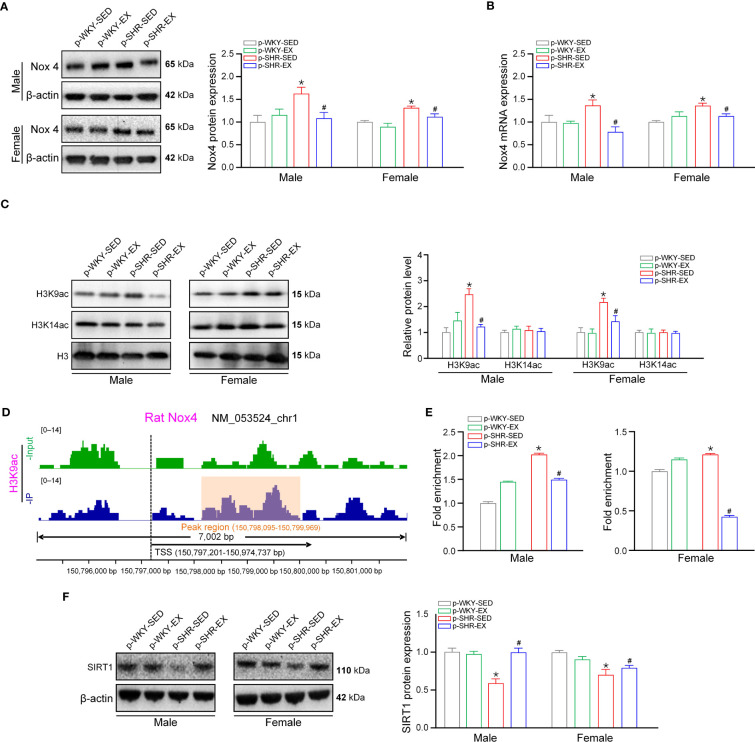
Maternal exercise induces H3K9 deacetylation of Nox4 promoter via SIRT in fetal mesenteric arteries. **(A)**, Representative immunoreactive bands and quantification analysis of Nox4 protein expression in mesenteric arteries from male and female offspring (n=6 samples per group). **(B)** Bar plot of Nox4 transcript levels in fetal mesenteric arteries (n=6 samples per group). **(C)** Representative immunoreactive bands and quantification analysis of H3K9ac and H3K14ac protein expression in fetal mesenteric arteries. **(D)** ChIP-seq detecting Peak of H3K9ac binding to the promoter region of Nox4 in fetal mesenteric arteries. **(E)** ChIP-PCR analysis showing acetylation level of H3K9ac in the promoter of Nox4 in fetal mesenteric arteries (n=3 samples per group). **(F)** Representative immunoreactive bands and quantification analysis of SIRT1 protein expression in fetal mesenteric arteries (n=6 samples per group). * *P*<0.05 compared with p-WKY-SED; ^#^ *P*<0.05 compared with p-SHR-SED.

Nox4 is a generating enzyme of ROS expressed in the endothelium, levels of which increase is involved in the pathophysiology of endothelial dysfunction (12). We observed that the NADPH oxidase inhibitor apocynin pre-incubation eliminated the difference of endothelium-dependent relaxation by ACh in mesenteric arteries between p-WKY-SED and p-SHR-SED or between p-SHR-SED and p-SHR-EX offspring (**Figures 5D, E**, both *P*>0.05). These results suggest that alteration of Nox4 in endothelial cells may result in impaired endothelium-dependent vasodilatation in hypertensive offspring and also play a pivotal role in ameliorating endothelial dysfunction in mesenteric arteries from SHR offspring by maternal exercise. As shown in **Figure 6A**, Nox4 protein level was significantly increased in mesenteric arteries from p-SHR-SED compared with p-WKY-SED in offspring at 3M (male: p-WKY-SED=1.00 ± 0.02 *vs.* p-SHR-SED: 1.63 ± 0.14, female: p-WKY-SED=1.00 ± 0.03 *vs.* p-SHR-SED: 1.32 ± 0.03; both *P*<0.05), whereas maternal exercise decreased Nox4 protein level by ~33% and ~6% in male and female SHR offspring, respectively. Consistent with this, the Nox4 mRNA expression of mesenteric arteries from p-SHR-SED fetuses were approximately 1.4-fold higher than p-WKY-SED fetus, while maternal exercise during pregnancy reduced this elevation by ~38% and ~19% in p-SHR-EX fetuses, when compared to SHR-SED male and female fetuses, respectively (*P*<0.05, **Figure 6B**).

To investigate further the mechanism of the upregulation of the Nox4 gene in fetal mesenteric arteries, we first investigated multiple major histone modifications in mesenteric arteries and found that H3K14ac were unchanged among groups, whereas H3K9ac was upregulated in p-SHR-SED and downregulated by maternal exercise (**Figure 6C**). Because H3K9ac is associated with transcriptional activation (25), and Nox4 was upregulated in p-SHR-SED offspring, we focused on studying H3K9ac. Using H3K9ac chromatin immunoprecipitation followed by sequencing, we found that Nox4 showed increased H3K9ac peak at promoter region (**Figure 6D**). To confirm the ChIP-seq results, we measured the enrichment of H3K9ac at Nox4 promoter region using anti-H3K9ac antibody in ChIP-qPCR experiment. We found the H3K9ac occupancy on Nox4 promoter was increased nearly 2.0-fold and 1.2-fold in mesenteric arteries from p-SHR-SED male and female fetuses, whereas was significantly decreased by ~26% and ~65% in p-SHR-EX male and female fetuses after maternal exercise (*P*<0.05, **Figure 6E**). SIRT1 is a major regulator of H3K9 acetylation, and several studies have shown that SIRT1 deacetylates the H3K9ac in the vasculature (26). We therefore focused on SIRT1, and observed SIRT1expression was reduced in mesenteric arteries from p-SHR-SED male and female fetuses (~59% and ~71% of p-WKY-SED, *P*<0.05), but enhanced by maternal exercise in p-SHR-EX male and female fetuses (~1.7-fold and 1.2-fold of p-SHR-SED, *P*<0.05; **Figure 6F**). These results suggested that SIRT1 mediates the decreased deposition of H3K9ac on Nox4 gene promoter to its gene-repressive effect during fetal development and contributed to the persistent downregulation of Nox4 in hypertensive offspring.

## Discussion

**Figure 7:**
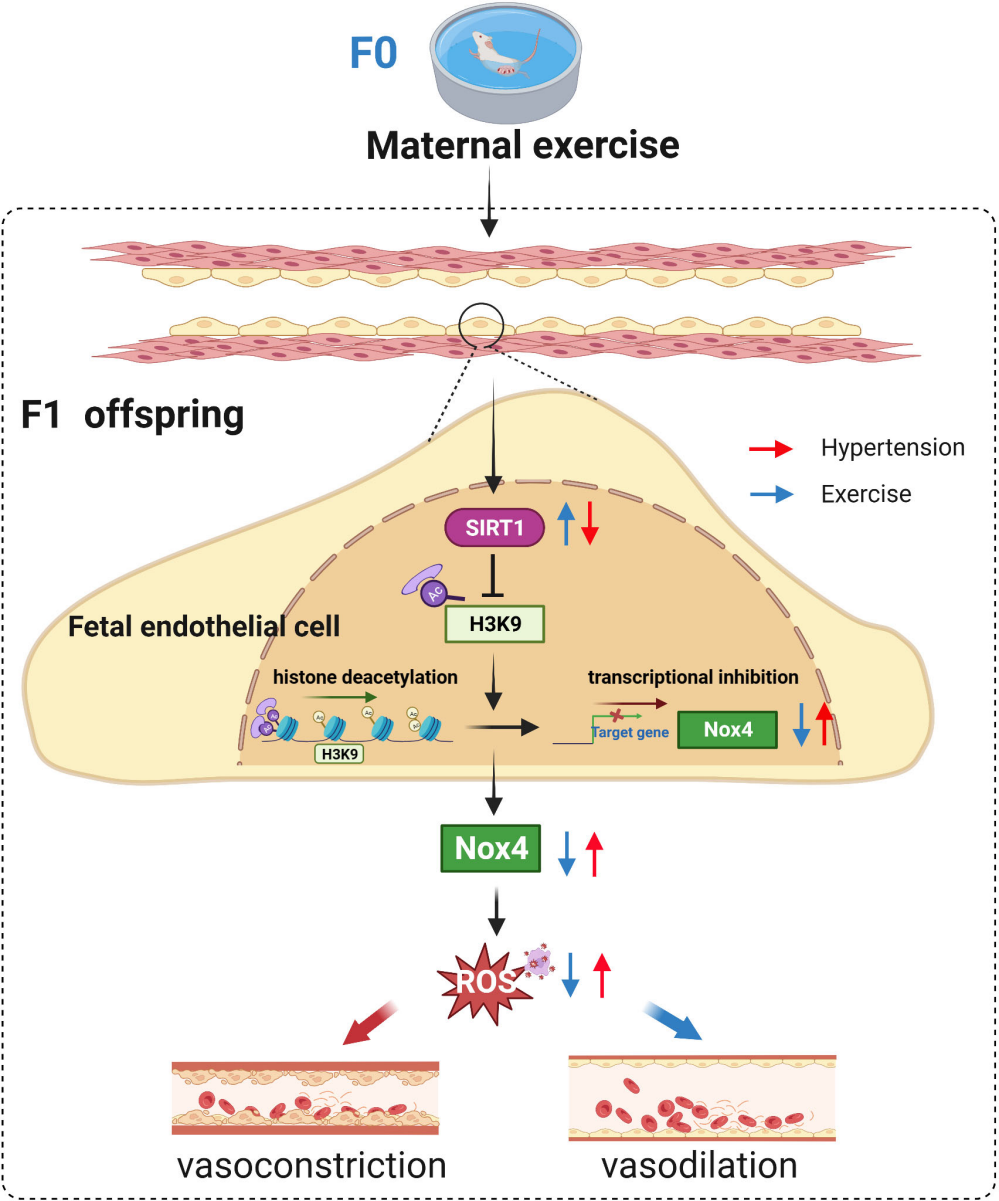
Proposed model for maternal exercise-induced repression of Nox4 via SIRT1-regulated H3K9ac to prevent vascular oxidative stress and endothelial dysfunction in SHR offspring.

Maternal exercise could have an important influence on the development and health of their offspring across generations (27). Up to now, the impacts of exercise during pregnancy on fetal development in hypertension, and its long-term effects on vascular endothelial health of hypertensive offspring are unknown. Our data show that maternal exercise can improve endothelium-dependent vasodilation in both male and female hypertensive offspring, which is associated with SIRT1-repressed Nox4 transcription by deacetylating histone H3K9 in mesenteric arteries during fetal development (**Figure 7**).

Epidemiological evidences have shown that systolic and diastolic pressures were strongly related to placental weight (systolic pressure rose by 15 mmHg as placental weight increased from ≤ 1 lb to > 1.5 lb) and birth weight (systolic pressure fell by 11 mmHg as birth weight increased from ≤ 5.5 lb to > 7.5 lb) in both sexes (28). Here, we found that the SHR fetuses exhibit lower fetal weight and placental insufficiency (fetal/placental weight), which may influence the later development of hypertension (29). Consistent with our results, Barrientos reported that SHR displayed a reduction of placental mass and signs of placental insufficiency (i.e. hypertrophy and reduced branching morphogenesis of the labyrinth layer), associated with decreased offspring weights and increased cephalization index indicating asymmetric fetal growth retardation, when compared with their normotensive genetic controls (30). Exercise during pregnancy has multiple effects on the fetoplacental development, including fetal growth, placenta-oriented cytokines, and placental mitochondrial biogenesis, nutrient transport and vascularization (18, 19, 27, 31, 32). As expected, exercise during pregnancy ameliorated fetal growth in SHR offspring, which may attribute to exercise-caused an acute reduction in oxygen, and eliciting compensatory increased nutrient delivery to the placental site (32, 33). Adverse environment exposure during pregnancy, including hypoxia, nicotine and high fat diet, is regarded as one of the causes of hypertension (34). In contrast, developmental plasticity also implies that an intervention during early development, such as maternal exercise, can prevent or delay hypertension in adult life (17). We showed maternal exercise reduced the blood pressure in male SHR offspring, but not in female. This different effect is agreement with other studies that insults during early life program sex differences in adult blood pressure and cardiovascular risk (35–37). The sex differences in blood pressure profiles in offspring may be related to differences in estrogen concentrations which estrogen improves vascular tone by decreasing smooth muscle cell proliferation or endothelium derived hyperpolarizing factor (NOS or cyclooxygenase-1) to protect vascular function in female (37, 38), which requires further investigation. Oxidative stress has been linked to increased blood pressure and emerged as a common process contributing to vascular dysfunction in a variety of pathological conditions, such as diabetes, atherosclerosis, and hypertension (5). The upregulation of plasma oxidative product MDA, meanwhile the reduction of antioxidant enzymes GSH-Px and SOD activity, and decreased NO production support that the oxidant capacity exceeded the antioxidant capacity in SHR offspring, may contribute in part to oxidative stress in hypertension. Maternal exercise altered these oxidative stress biomarkers, leading to the improvement of oxidative stress status in SHR offspring. These results are similar to those observed in adult hypertensive rats subject to aerobic exercise (39–41). Numerous studies have demonstrated the role of oxidative stress as facilitator of different processes leading to vascular remodeling in different hypertensive models including preeclampsia (41–44). We found that mesenteric arteries from SHR offspring showed smaller incremental distensibility and enhanced vascular stiffness, Maternal exercise normalized the increased vascular stiffness and improved the reduced vascular distensibility in male offspring, but only decreased wall/lumen ratio of mesenteric arteries from female SHR offspring. These findings suggest that maternal exercise induces a different structural vascular adaptation to restore abnormal vessel structure in mesenteric arteries between male and female SHR offspring.

In addition to improved morphology of vessels, maternal exercise restores endothelial function in mesenteric arteries of SHR offspring. Endothelium-dependent vasodilatation was impaired in mesenteric arteries from SHR offspring, as previously described in SHR models (41, 45). Endothelium-independent vasodilatations in response to SNP were comparable, indicating the impairment of vascular function in SHR offspring was more specific to the endothelium. More importantly, maternal exercise significantly ameliorated the impaired ACh vasodilation without affecting SNP-induced vasodilation in both male and female SHR offspring. We found that endothelium-dependent vasodilatation was in part mediated by NO in mesenteric arteries. A loss of NO-mediated vasodilatations in mesenteric arteries of SHR offspring was observed, which is consistent with previous report in DOCA-salt hypertension mice (46). Maternal exercise prevents the loss of NO-mediated vasodilatations in hypertensive offspring. It is unlikely that down-regulation of eNOS protein expression is responsible for reduced NO-mediated vasodilatation because eNOS expression was unaltered in mesenteric arteries from SHR offspring. In addition, this difference appeared to be due to alteration in the phosphorylation status of eNOS, although some studies in endothelial cells may ascribe it to eNOS encouplin (2). Consistent with the above results, endothelial ROS production assessed by DHE fluorescence in mesenteric arteries was significantly increased in SHR offspring, which was inhibited by maternal exercise. Activation of NADPH oxidase and dysfunction of mitochondria are considered major contributors to ROS in endothelial cells (11). NADPH oxidase inhibitor, apocynin, abolished the difference of the ACh-induced relaxation in offspring, suggesting that maternal exercise improves endothelial function of mesenteric arteries in SHR offspring by inactivate NADPH oxidase. Here, we found Nox4 expression was increased in mesenteric arteries from SHR offspring, which was reduced by maternal exercise. These findings suggest that upregulation of Nox4 in the endothelium contributes to vascular dysfunction in SHR offspring, at least in part, through a burst of ROS generation. However, maternal exercise prevents the Nox4 elevation and alleviates ROS generation, restores vascular function in hypertensive offspring.

Various types of exogenous exposure during perinatal development can influence epigenetic modifications, which have gained considerable attention in the pathogenesis of hypertension (47). Histone modification is typical epigenetic modification regulating transcription of target gene expression both positively and negatively (48). Lipopolysaccharides exposure during pregnancy triggered oxidative stress that upregulated KDM3B (histonelysine demethylase 3B) in the oocytes of first-generation female rats, reducing H3K9me2 modification, resulting in transgenerational upregulation of Rac1 gene in offspring (48). We focused on an upregulated histone modification, H3K9ac, a negative regulator of the Nox4 expression. We used chromatin immunoprecipitation sequencing and qPCR to validate the causal role of the high H3K9ac occupancy in the upregulation of the Nox4 gene in mesenteric arteries in SHR offspring. SIRT1, a highly conserved NAD+-dependent deacetylase, is one member of the SIRT family of class III HDACs involved in the development of endocrine and metabolic diseases (22, 26). Furthermore, we identified SIRT1, the histone deacetylase of H3K9ac, increasing the H3K9ac in mesenteric arteries. Maternal exercise drives the SIRT1-regulated H3K9ac, thereby decreasing H3K9ac level and repressing Nox4 expression in mesenteric arteries from fetal SHR.

Taken together, this study showed that maternal exercise can prevent vascular oxidative stress and improve endothelial function in hypertensive offspring, and that the underlying mechanism is related to histone modification and transcriptional repression of Nox4 in fetal mesenteric arteries. This work provides an epigenetic mechanism that underlie the effects of maternal exercise on offspring vascular health and could potentially uncover an approach to prevent the transmission of hypertension across generations.

## Data availability statement

The raw data supporting the conclusions of this article will be made available by the authors, without undue reservation.

## Ethics statement

The animal study was reviewed and approved by Animal Care and Use Committee of the Beijing Sport University.

## Author contributions

YZ designed and conducted the experiments, analyzed the data and wrote the manuscript. MS conducted experiments, analyzed data and wrote the manuscript. XD, HS, and FQ conducted experiments. LS conceived and designed the studies, and interpreted the data. All authors contributed to the article and approved the submitted version.
